# COMPARISON OF SELF-RATED HEALTH, FRAILTY-INDEX, AND MULTIMORBIDITY AS HEALTH INDICATORS

**DOI:** 10.1093/geroni/igad104.3241

**Published:** 2023-12-21

**Authors:** Pauliina Halonen, Linda Enroth, Tuija Jääskeläinen, Seppo Koskinen, Marja Jylhä, Laura Kananen

**Affiliations:** Tampere University, Tampere, Pirkanmaa, Finland; Tampere University, Tampere, Pirkanmaa, Finland; Finnish Institute for Health and Welfare, Helsinki, Uusimaa, Finland; Finnish Institute for Health and Welfare, Helsinki, Uusimaa, Finland; Tampere University, Tampere, Pirkanmaa, Finland; Tampere University, Tampere, Pirkanmaa, Finland

## Abstract

Self-rated health (SRH) predicts mortality and other adverse events (e.g. hospitalizations, intensive care), and reflects clinical biomarker levels even in individuals without diseases. We hypothesize SRH captures information relevant to clinical decision-making, with minimal effort, and might be used prior to more intensive and focused health assessments. To explore the clinical utility of SRH, we 1) analysed its overlap with clinical health assessments, multimorbidity, and Rockwood frailty-index (FI), and 2) compared their performances in mortality prediction. Our data came from two population-based cohort studies in Finland: Health 2000 Survey (H2000: n=6,329, aged 30-97 years) and National FinHealth 2017 Study (FH17: n=4,105, aged 30-98 years). Cox regression adjusted for age and sex, and Harrel’s C-index were used to assess the performances of continuous SRH, FI, and multimorbidity in 4-years-all-cause mortality prediction. Of those with fair/poor SRH, 85% in H2000 and 91% in FH2017 were also frail (FI≥0.25), and 91% and 81% had at least two diseases, respectively. Baseline SRH (H2000: HR[95CI]=3.14[2.10-4.69], FH17: HR[95CI]=4.57[2.37-8.83]), FI (H2000: HR[95CI]=1.40[1.28-1.53], FH17: HR[95CI]=1.54[1.32-1.80]) and number of diseases (H2000: HR[95CI]=1.18[1.10-1.27], FH17: HR[95CI]=1.18[1.03-1.35]) predicted death, and their C-indices were 0.879, 0.884, 0.877 in H2000 and 0.797, 0.800, 0.779 in FH17, respectively. SRH is concordant with clinical health indicators, such as multimorbidity and FI, and predicts mortality equally well in community-dwelling adults. The analyses are ongoing and we will further assess the utility of SRH in other cohorts, using other outcomes, and clinical indicators from registers, and perform age-, sex- and disease-stratified analyses.

